# Effects of the mitochondria-targeted antioxidant SkQ1 on lifespan of rodents

**DOI:** 10.18632/aging.100404

**Published:** 2011-12-11

**Authors:** Vladimir N. Anisimov, Maxim V. Egorov, Marina S. Krasilshchikova, Konstantin G. Lyamzaev, Vasily N. Manskikh, Mikhail P. Moshkin, Evgeny A. Novikov, Irina G. Popovich, Konstantin A. Rogovin, Irina G. Shabalina, Olga N. Shekarova, Maxim V. Skulachev, Tatiana V. Titova, Vladimir A. Vygodin, Mikhail Yu. Vyssokikh, Maria N. Yurova, Mark A. Zabezhinsky, Vladimir P. Skulachev

**Affiliations:** ^1^ Petrov Institute of Oncology, St. Petersburg 197758, Russia; ^2^ The Wenner-Gren Institute, Stockholm University, SE-106 91 Stockholm, Sweden; ^3^ Institute of Mitoengineering, Lomonosov Moscow State University, Moscow 119991, Russia; ^4^ Shemyakin and Ovchinnikov Institute of Bioorganic Chemistry, Moscow, Russia; ^5^ Institute of Cytology and Genetics, Novosibirsk 630090, Russia; ^6^ Institute of Systematics and Ecology of Animals, Novosibirsk 630091, Russia; ^7^ Severtsev Institute of Ecology and Evolution, Moscow 119071, Russia; ^8^ Belozersky Institute of Physico-Chemical Biology, Lomonosov Moscow State University, Moscow 119991, Russia; ^9^ Faculty of Bioengineering and Bioinformatics, Lomonosov Moscow State University, Moscow 119991, Russia

**Keywords:** Aging, mitochondria-targeted antioxidant, SkQ1, lifespan

## Abstract

The effect of the mitochondria-targeted, plastoquinone-containing antioxidant SkQ1 on the lifespan of outbred mice and of three strains of inbred mice was studied. To this end, low pathogen (LP) or specific pathogen free (SPF) vivaria in St. Petersburg, Moscow, and Stockholm were used. For comparison, we also studied mole-voles and dwarf hamsters, two wild species of small rodents kept under simulated natural conditions. It was found that substitution of a LP vivarium for a conventional (non-LP) one doubled the lifespan of female outbred mice, just as SkQ1 did in a non-LP vivarium. SkQ1 prevented age-dependent disappearance of estrous cycles of outbred mice in both LP and non-LP vivaria. In the SPF vivarium in Moscow, male BALB/c mice had shorter lifespan than females, and SkQ1 increased their lifespan to the values of the females. In the females, SkQ1 retarded development of such trait of aging as heart mass increase. Male C57Bl/6 mice housed individually in the SPF vivarium in Stockholm lived as long as females. SkQ1 increased the male lifespan, the longevity of the females being unchanged. SkQ1 did not change food intake by these mice. Dwarf hamsters and mole-voles kept in outdoor cages or under simulated natural conditions lived longer if treated with SkQ1. The effect of SkQ1 on longevity of females is assumed to mainly be due to retardation of the age-linked decline of the immune system. For males under LP or SPF conditions, SkQ1 increased the lifespan, affecting also some other system(s) responsible for aging.

## INTRODUCTION

During the last seven years our group has studied the possibility of retarding aging with a mitochondria-targeted antioxidant composed of plastoquinone conjugated with a penetrating cation, decyltriphenyl-phosphonium, where the positive charge is strongly delocalized over three aromatic substituents of the phosphorous atom. SkQ1 [10-(6'-plastoquinonyl) decyltriphenylphosphonium] was synthesized [1, 2] and tested on fungi, crustaceans, insects, fish, and mice. It was found to increase the median lifespan of these organisms, and it retarded, arrested, and in some cases even reversed development of many age-related pathological traits [1-8]^1^. (For theoretical background on this project, i.e. the concept of programmed aging, see references [1, 9-11]).

Mice are a classical subject of gerontological studies of mammals. In such studies, a certain laboratory strain of inbred mice kept under specific pathogen free (SPF) conditions is usually employed. This approach has been criticized, since inbred laboratory strains of mice, as well as SPF conditions, hardly mimic natural conditions [12-14]. Therefore, our experiments described in this paper were carried out on (i) several strains of inbred mice differing in lifespan, (ii) outbred mice, (iii) mice living in different low pathogen (LP) or SPF vivaria, (iv) mice living in a conventional (non-LP) vivarium, and (v) two species of wild rodents, i.e. mole-voles and Campbell dwarf hamsters, kept in outdoor cages. It was found that the lifespan of the animals and effect of SkQ1 on this parameter depended on genetic background, sex, and life conditions.

## RESULTS AND DISCUSSION

### Effects of SkQ1 on outbred mice

In preceding papers [1, 2, 4], it has been shown that female outbred SHR mice living in an old non-LP vivarium in St. Petersburg were short-lived (median lifespan about 300 days). They died mainly due to various infections, the mortality being age-dependent. In these experiments, the median lifespan was doubled by very low doses of SkQ1 (5 nmol SkQ1/kg per day) which greatly decreased the infection-related mortality. SkQ1 also changed the main reason for death: In the presence of SkQ1, mammary carcinomas, rather than infections, were responsible for the majority of deaths. This looked as if the SkQ1-treated females lived so long that they eventually attained the age when carcinomas appeared (for SHR mice, this critical age was ≥ 400 days [3]). It is noteworthy that SkQ1 did not affect the lifespan of short-lived inbred HER-2 mice prone to mammary carcinoma and dying exclusively due to development of this kind of cancer [3]^2^. Strong increase in the lifespan of mice in the non-LP vivarium is consistent with our observations that in OXYS rats, SkQ1 retards an age-related decline of the immune system. We found that age-dependent involution of thymus and spleen follicles, i.e. tissues producing T- and B-lymphocytes, respectively, was decelerated by SkQ1 [4, 8, 15], and that SkQ1 prevented the age-linked decrease in total lymphocyte content in the blood of mice [16]. It should be stressed that SkQ1 possesses no antibiotic activity. Rather, it is favorable for survival of bacteria under conditions of oxidative stress (I. V. Manukhov et al., in preparation). Moreover, SkQ1 was without any effect on mortality of young mice infected by tuberculosis (A. S. Apt, personal communication).

**Figure 1:**
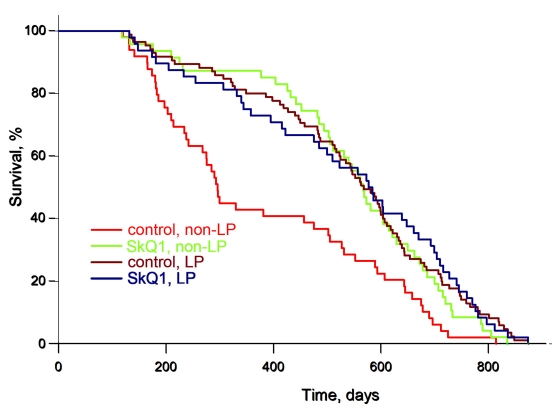
Low pathogen (LP) conditions extend the lifespan of female outbred SHR mice to the same degree as an optimal concentration of SkQ1. A total of 155 mice living in the LP vivarium of the Institute of Oncology in St. Petersburg were used. For comparison, data of experiments with 200 SHR mice living in the non-LP vivarium of the same institute and already published [3] are shown. Where indicated, the mice received 5 nmol SkQ1/kg per day (which was added to the drinking water) during their entire life.

**Figure 2:**
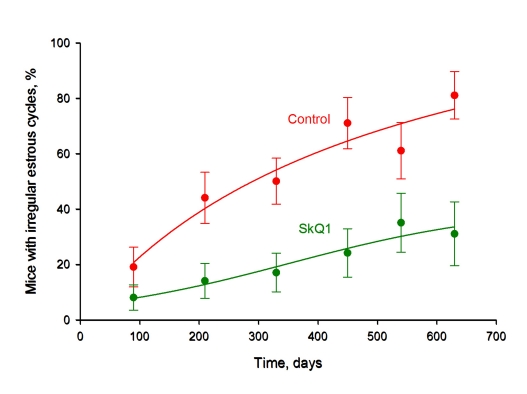
SkQ1 prevents age-dependent disappearance of regular estrous cycles in outbred SHR mice living in the LP vivarium of the Institute of Oncology, St. Petersburg. A total of 155 mice were studied. Where indicated, 5 nmol SkQ1/kg per day was administered. *, *p* < 0.05.

We now present results on females from the same colony of outbred SHR mice as in the preceding study [1, 2, 4] but living in a LP vivarium (Figure 1) (for comparison, data from the earlier experiments performed under non-LP conditions [3] are shown in the same figure). The median lifespan was doubled in the LP vivarium, independently of whether the mice were SkQ1 treated or not. This does not mean that SkQ1 was without any influence on the mice under LP conditions. For example, SkQ1 prevented the age-dependent disappearance of estrous cycles in females, a typical trait of aging of these animals (Figure 2).

Similar effects were revealed when another geroprotector, metformin [17], was used instead of SkQ1. The primary difference between the two compounds was that metformin was very much less efficient (100 mg metformin/kg per day was needed, data not shown; in the case of SkQ1, as little as 3 μg/kg per day was sufficient).

### Effects of SkQ1 on inbred mouse strains

**Figure 3:**
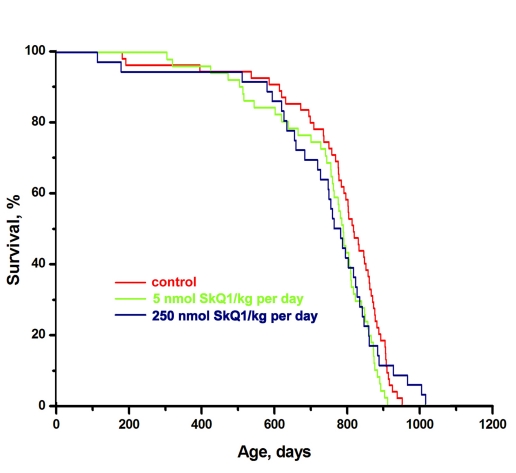
Effect of SkQ1 on the lifespan of females of long-lived 129/sv mice in the LP vivarium of the Institute of Oncology, St. Petersburg. A total of 142 mice were studied. Differences between the curves are not statistically significant.

In further experiments, longer-lived inbred female 129/sv mice were studied in the same LP vivarium (St. Petersburg). The median lifespan was as long as 830 days (cf. 600 days for the outbred SHR mice under the LP conditions). Neither 5 nor 250 nmol SkQ1/kg per day had any statistically reliable effect on the longevity of these mice (Figure 3) or on “healthspan” (monitored by measuring regularity of estrous cycles). In the 129/sv mice, the estrous cycles were regular for as long as 20 months even without any geroprotector (not shown). A possible explanation for these observations might be that the 129/sv strain has lost an SkQ1-inhibitable aging program.

**Figure 4:**
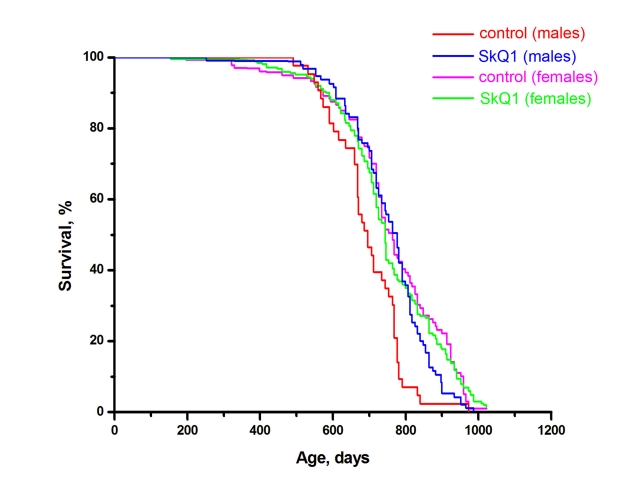
Effect of SkQ1 on the lifespan of male and female BALB/c mice. The specific pathogen free (SPF) vivarium of the Shemyakin and Ovchinnikov Institute of Bioorganic Chemistry in Moscow was used. During the first year, 10 males were housed in each cage. Thereafter they were individually housed. Cages with females contained 10 animals during their entire life. A total of 440 mice were studied. Two SkQ1 concentrations were used, 1 and 30 nmol/kg per day. Results of the two SkQ1-receiving groups were combined. For males, *p* < 0.05 for the SkQ1-induced increase in lifespan.

**Figure 5:**
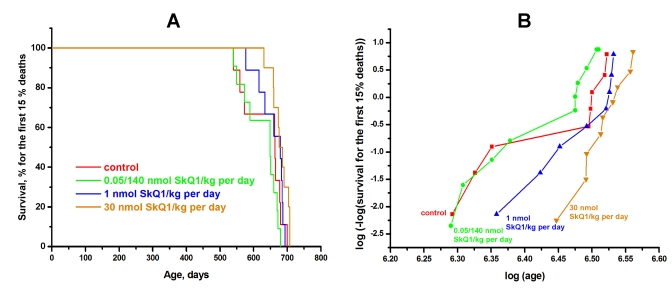
SkQ1 increases in dose-dependent manner the lifespan of female BALB/c mice if survival for the first 15% of deaths is considered. Three SkQ1 concentrations were used (nmol SkQ1/kg per day): (i) 1, (ii) 30, and (iii) 0.05 during the first 595 days and 140 for subsequent days. In B, *p* was <0.05 for 1 and 30 nmol SkQ1/kg per day compared with the control or 0.05/140 nmol SkQ1/kg per day. The vivarium used was the same as in Figure 4.

Inbred male and female BALB/c mice were studied in the SPF vivarium in Moscow. SkQ1 did not influence the *female* median lifespan. However, a statistically significant effect was observed for the *males*. The median lifespan of untreated males was 690 days, i.e. shorter than that of females (770 days). SkQ1 increased the lifespan of the males to that of the females (Figure 4). More detailed analysis of the BALB/c strain females revealed a statistically reliable effect of SkQ1 upon survival curves in the region of in the first 15% of deaths (Figure 5A). This effect was especially clear when log (–log of survival) values were plotted against log of the age (Figure 5B). Moreover, SkQ1 was found to retard development of such trait of senescence as increase with age of heart mass [18]. In females, SkQ1 attenuated this effect of aging by almost 50% (Table 1). A similar effect of SkQ1 was observed in males (data not shown).

**Table 1:** SkQ1 retards age-dependent increase in heart mass of female BALB/c mice Statistical significant *p* < 0.05 was shown for combined group of old mice receiving 1 or 30 nmol SkQ1/kg per day compared with the control group (2) of old mice.

Group	Age, months	nmol SkQ1/kg per day	Number of animals	Heart mass, mg (M±m)
1	3	-	15	115 ± 3
2	24	-	14	157 ± 9
3	24	1	15	139 ± 4
4	24	30	15	139 ± 4
5	24*	0.05/140	14	146 ± 6

* Mice received 0.05 nmol SkQ1/kg per day during first 595 days of life and 140 nmol SkQ1/kg per day during the next 130 days.

Another line of inbred mice, C57Bl/6, was studied under SPF conditions in a Stockholm vivarium. In this study the animals were individually housed. The median lifespan was similar in the males and females (740 and 773 days, respectively)^3^. Life-long treatment with SkQ1 again did not affect the median lifespan of females but increased it to 937 days in males (I. G. Shabalina, B. Cannon, J. Nedergaard et al., in preparation). In the same experiments, it was shown that SkQ1 did not affect the food intake of the animals, which was consistent with our previous observation on SHR mice [21]. Aging did not influence the total leukocyte content in the C57Bl/6 mice but strongly increased the number of neutrophils and monocytes, while levels of eosinophils and lymphocytes decreased. SkQ1 treatment attenuated these changes (I. G. Shabalina, B. Cannon, J. Nedergaard et al., in preparation). Similar data have been reported for BALB/c mice [16].

### Effects of SkQ1 on wild rodents

In another series of experiments, we studied the lifespan of two species of wild rodents housed in outdoor cages or kept under conditions that simulated natural seasonal changes in temperature and illumination.

**Figure 6:**
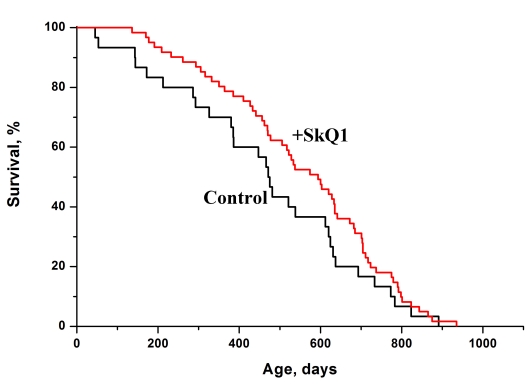
SkQ1 increases the lifespan of females of a wild rodent, the dwarf hamster *Phodopus campbelli* living in outdoor cages. A total of 91 animals were studied. Statistical significance *p* < 0.05. Two concentrations of SkQ1 (5 and 50 nmol/kg per day) were used. The data of two SkQ1 groups were combined.

The dwarf hamster *Phodopus campbelli* was one of these species. In Figure 6 survival curves of the female hamsters are given. It is seen that SkQ1 increased the lifespan of this rodent.

**Figure 7:**
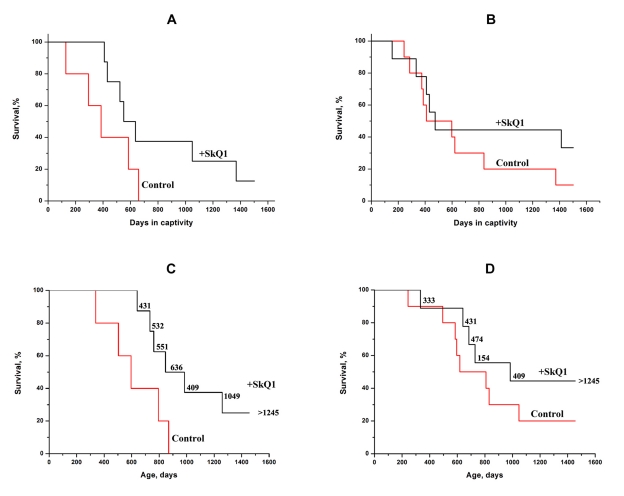
SkQ1 (50 nmol/kg per day) increases the lifespan of a wild rodent, the mole-vole *Ellobius talpinus*, under simulated natural conditions. A total of 32 animals were used in the experiments. **A**, **C**, males; **B**, **D**, females. Statistical significance *p* < 0.05 (SkQ1 versus control). Figures near the SkQ1 curves (C, D), duration of the SkQ1 treatment (days). Age of animals (**C**, **D**) was determined *post mortem* by measuring the parameters of the root of the 1st mandibular molar.

Another species studied was the mole-vole *Ellobius talpinus*. The animals were captured in a taiga forest (Novosibirsk region), so their age could be estimated by means of *post mortem* methods only. Some of the SkQ1-treated animals were so long-lived that we had to present the data in two ways, namely by survival plotted against (i) the time spent in captivity (for all the animals studied, Figures 7A, B), and (ii) the age (for animals who died during the study, Figures 7 C, D). In the first case, the survival curves for SkQ1-treated mole-voles looked very unusual: they showed, in fact, two distinct phases. The first phase (until days 640 and 470 in captivity for males and females) when 50-60% of the animals had died, and the second phase when almost no deaths occurred in the SkQ1 groups (for males, only two animals died between days 640 and 1,500 in captivity; for females, one animal died between days 470 and 1,500). For the control (non-treated) mole-voles, the last male died on day 660 in captivity, whereas one of females survived at least to day 1,500.

The age of the mole-voles was estimated *post mortem* (roots of the first mandibular molars were studied). The number of animals in this case was slightly less than the total number studied in the experiment because some corpses were destroyed by the neighbors living in the same cage. The median lifespan was significantly increased by SkQ1, the effect being stronger for males. A *post mortem* study revealed only one case where a tumor was identified in the dead mole-vole, whereas in mice of the same age the great majority of dead animals contained tumors.

In contrast to other experiments described above, male and female mole-voles were kept together. This allowed us to observe a striking effect of SkQ1 on such senescence trait as the loss with age of reproductive ability. As shown in Table 2, in the control group fecundity strongly decreased in the second year. This was not the case in the SkQ1-treated group, who continued to be productive even in the third year of life. The unusual biphasic shape of the survival curves of mole-voles (Figure 7) should be discussed in more detail. We suggest that the reason for this is that some animals received SkQ1 at a younger age than others. This seemed to be quite likely since among the mole-voles caught in the Siberian taiga were animals of various ages. As previously shown in our group [3, 4], one week-long SkQ1 treatment prolonged the lifespan of *Drosophila* if the treatment was carried out during the first week of life, but it was ineffective when given for the same period in the middle of life. Assuming SkQ1 is an inhibitor of the execution of the aging program, we speculate that it fails to induce a strong increase in lifespan of mice since the SkQ1-treated animals die, after all, as a result of cancer. If cancer is a very rare reason for death of mole-voles, a large increase in the lifespan by SkQ1 becomes possible. This possibility was confirmed when *post mortem* estimation of age of dead mole-voles was carried out. All the SkQ1-treated animals who died during first two years in captivity started to receive the antioxidant since the 2^nd^ year of the life (Figures 7C,D). It is remarkable that a small number of mole-vole females showed a long-lived phenotype even without SkQ1 (Figures 7B, D). This fact seems to indicate that these animals have at their disposal a mechanism to switch off the aging program. In such a state, mole-vole females resemble another mole-like rodent, the long-lived naked mole-rats that are also cancer-resistant and show no age-dependent increase in the probability of death [22-29]. Remarkably, mole-voles, like mole rats, are eusocial animals forming communities composed of 12-25 individuals (for mole-rats, these values are larger) [30].

**Table 2:** Effect of SkQ1 on fecundity of mole-vole *Ellobius talpinus*

Year	No SkQ1			With 50 nmol SkQ1/kg per day		
	Number of females	Number of litter	Number of newborn animals	Number of females	Number of litters	Number of newborn animals
2008	5	7	18	6	9	25
2009	3	1	2	4	5	16
2010	2	-*	-*	4	5	10

* No males survived in the cage

In conclusion, the mitochondria-targeted antioxidant SkQ1 prolongs the lifespan of outbred mice, dwarf hamsters, and mole-voles kept in a conventional vivarium or outdoor cages. Under low pathogen or specific pathogen free conditions, female outbred mice lived as long as in a conventional vivarium with SkQ1, and the antioxidant fails to further increase the lifespan of animals who die because of cancer rather than infections. However, in this case SkQ still prolonged the healthspan, preventing the age-dependent disappearance of estrous cycles. Females of two strains of inbred mice (BALB/c and 57B1/6) kept in SPF vivaria were similar to outbred mice. For males, SkQ1 caused some increase in lifespan of these strains even under SPF conditions. Inbred 129/sv mice were the longest-lived among the studied strains and retained estrous cycles for a long time even without the antioxidant, which did not affect their lifespan. SkQ1 prolonged the median lifespan of dwarf hamsters and mole-voles under non-LP conditions, the effect being especially great in the case of mole-voles treated with SkQ1 from an early age. Cancer was found be absent from the main reasons for the death of mole-voles. Apparently, the possibility to increase the lifespan with SkQ1 in other studied animals was limited by death caused by cancer. Thus, the results of our analysis of effects of SkQ1 on the lifespan and healthspan of rodents are consistent with the assumption that this antioxidant retards the operation of an aging program.

## METHODS

In this study we used laboratory outbred SHR mice^4^ and three strains of inbred mice differing greatly in their position on the mouse family tree [31], i.e. 129/sv, BALB/c, and C57Bl/6. The mice were housed in an LP vivarium at the Petrov Institute of Oncology (St. Petersburg) and in the SPF vivaria of the Shemyakin and Ovchinnikov Institute of Bioorganic Chemistry (Moscow) and the Wenner-Gren Institute (Stockholm). In some of these studies, the estrous cycles of females were followed every three months as described earlier [32]. Complete *post mortem* autopsies were performed for histological analysis of tumors and non-tumor pathologies [33]. SkQ1 was administered by addition to the drinking water.

Two species of small wild rodents were studied, the mole-vole *Ellobius talpinus* (Novosibirsk region, Siberia) and the dwarf hamster *Phodopus campbelli* (Mongolia).

The *mole-voles*, small (45-50 g) subterranean rodents, were kept under simulated natural conditions, the animals being housed in cages (two males and two females per cage) in a non-sterile room where temperature and light/dark regimes roughly corresponded to the natural situation in nesting places of this rodent, i.e. 15-20°C, 16 h: 8 h light: dark periods (April-November) and 10-15°C, constant darkness (November-April). These regimes were chosen since in the summer the mole-voles form nests in the ground at the depth of 20- 50 cm and rather often visit the surface, while in the winter they are always in deeper nests (1.5-3 m) where temperature usually varies from 10 to 15°C. The control and the SkQ-treated groups included 16 animals each. The SkQ1-treated animals received 50 nmol SkQ1/kg per day with food. As food, carrots and standard mouse chow (Laboratorsnab, Ltd., Moscow) were used.

*Female dwarf hamsters*, 30-40 g rodents, were kept in outdoor cages (6-8 animals per cage). As food a mixture of standard mouse chow (see above), bread, seeds of oats, millet and sunflower, cabbage, beets, and carrots *ad libitum* were provided. In the summer or the winter, the food was supplemented with curd and boiled meat once or twice per month, respectively. In the summer, 5 or 50 nmol SkQ1/kg per day was added to the drinking water. In the winter, when the animals received water exclusively from vegetables, they received SkQ1 *per os* as drops of an aqueous solution. The control, 5 nmol SkQ1, and 50 nmol SkQ1 groups consisted of 30, 31, and 30 animals, respectively. (For description of mole-voles and dwarf hamsters in more detail, see references [30] and [34], respectively).

Statistic analyses were carried out according to the Kaplan-Meier method [35].

All experiments on animals were carried out in accordance with the animal care regulations of the Shemyakin and Ovchinnikov Institute of Bioorganic Chemistry (Moscow), Wenner-Gren Institute (Stockholm), Institute of Cytology and Genetics (Novosibirsk), and the Severtsev Institute of Ecology and Evolution (Moscow).

## Footnotes

1 Refs. 1-3, 6-8 as well as our other papers published in Biochemistry (Moscow), English version, and quoted in this article are available in Internet (see http://protein. bio.msu.ru/biokhimiya).

2 At the same time, SkQ1 significantly prolonged the lifespan of p53^−/−^ mice prone to lymphomas [7].

3 Longer lifespan of males of C57Bl/6 than of BALB/c might be due to the fact that the BALB/c males were kept collectively during the first year of the life. Under such conditions, males are fighting and such a stress may shorten their life. This fact might explain shorter lifespan of males than females of the same strain [19, 20].

4 Abbreviation SHR in this case does not mean hypertensive (cf. hypertensive SHR strain of rats).

## Abbreviations

ROS: reactive oxygen species
SkQ1: plastoquinonyl-10(6′- decyltriphenyl)phosphonium
LP: low pathogen
SPF: specific pathogen free
